# White sweet potato ameliorates hyperglycemia and regenerates pancreatic islets in diabetic mice

**DOI:** 10.29219/fnr.v64.3609

**Published:** 2020-03-02

**Authors:** Chun-Kuang Shih, Chiao-Ming Chen, Viola Varga, Liang-Chen Shih, Peng-Ru Chen, Shu-Fang Lo, Lie-Fen Shyur, Sing-Chung Li

**Affiliations:** 1 School of Nutrition and Health Science, College of Nutrition, Taipei Medical University, Taipei, Taiwan; 2 Department of Food Science, Nutrition, and Nutraceutical Biotechnology, Shih Chien University, Taipei, Taiwan; 3 Department of Agronomy, Chiayi Agricultural Experiment Station, Taiwan Agricultural Research Institute, Chiayi, Taiwan; 4 Agricultural Biotechnology Research Center, Academia Sinica, Taipei, Taiwan

**Keywords:** white sweet potato, antidiabetic, antihyperglycemic, pancreatic islets, insulin sensitivity

## Abstract

**Background:**

White sweet potato (WSP) has many potential beneficial effects on metabolic control and on diabetes-related insulin resistance. The antihyperglycemic effects of Tainung No. 10 (TNG10), a variety of WSP in Taiwan, warrant investigation.

**Objective:**

To investigate the antidiabetic activity of WSP (*Ipomoea batatas* L. TNG10) and the mechanisms for interventions using whole leaves or tubers of WSP in diabetic mice.

**Design:**

Mice were co-administered with streptozotocin and nicotinamide to induce diabetes and then treated with an experimental diet including either 10% WSP tuber (10%-T) and 30% WSP tuber (30%-T) or 0.5% WSP leaf (0.5%-L) and 5% WSP leaf (5%-L). After 8 weeks’ treatment, their plasma glycemic parameters, lipid profiles, and inflammatory marker were analyzed. Their pancreases were removed for histopathologic image analysis; proteins were also extracted from their muscles for phosphoinositide 3-kinase pathway analysis.

**Results:**

The 30%-T or 5%-L mice had lower plasma glucose, insulin, glucose area under the curve (AUC), homeostatic model assessment of insulin resistance (HOMA-IR), alanine transaminase, triglyceride, and tumor necrosis factor alpha levels. In all diabetic mice, their Langerhans’s area was reduced by 60%; however, after 30% WSP-T or 5% WSP-L diets, the mice demonstrated significant restoration of the Langerhans’s areas (approximately 30%). Only in 5%-L mice, slightly increased expression of insulin-signaling pathway-related proteins, phosphorylated insulin receptor and protein kinase B and membrane glucose transporter 4 was noted.

**Conclusions:**

WSP has antihyperglycemic effects by inducing pancreatic islet regeneration and insulin resistance amelioration. Therefore, WSP has potential applications in dietary diabetes management.

## Popular scientific summary

- The 30%-T or 5%-L of WSP had lower plasma glucose, insulin, AUC, HOMA-IR, alanine transaminase, triglyceride, and tumor necrosis factor alpha in diabetic mice.
- The DM+30%-T or DM+5%-L of WSP had significantly restored Langerhans’s area by approximately 30%.
- The 5%-L of WSP increased insulin sensitivity possibly via the insulin-signaling pathway and reduce blood glucose levels in diabetic mice.

Diabetes is one of the most prominent public health concerns of the 21st century and is characterized by the effects of either increasing insulin resistance or impairing glucose tolerance (1). According to the latest global estimate from the International Diabetes Federation, 415 million people had diabetes in 2015, and this number is expected to reach 642 million by 2040 (2). Glucose homeostasis is maintained by the fine orchestration of insulin secretion and activity both for promoting glucose transport into muscle and adipocytes and for inhibiting liver glucose output. Defects in insulin signaling impair glucose utilization and are believed to be a critical factor in insulin resistance pathogenesis (3). Resistance to these effects of insulin is a classic pathogenic feature of obesity and type 2 (non-insulin-dependent) diabetes mellitus (T2DM) (4, 5). In T2DM, insulin resistance is initially compensated by increased secretion of insulin; however, this prolonged hyperinsulinemia leads to progressive β-cell exhaustion and degradation (6). The co-administration of streptozotocin (STZ) and nicotinamide (NA) can be used to induce T2DM animal models; this is because STZ causes pancreatic β-cell damage, whereas NA partially protects insulin-secreting cells against STZ damage (7–9).

The phosphoinositide 3-kinase (PI3K) signaling pathway, a signal transduction system downstream of an insulin receptor (IR), is a key factor in the translocation of the glucose transporter protein from intracellular compartments to plasma membrane (10). IRs are present in most mammalian cells, and insulin-IR binding results in the activation of several phosphorylation-dephosphorylation cascades (11). Autophosphorylation of the intracellular β-subunit of IRs activates tyrosine kinase, which catalyzes multiple IR substrate (IRS) protein phosphorylation (12). Disruption in IRS protein phosphorylation or impaired PI3K recruitment from the cytosol, which results in PI3K inactivation, causes insulin resistance, followed by diabetes (13). Protein kinase B (Akt; also called alpha serine/threonine protein kinase) activation by growth factors occurs in a PI3K-dependent manner. Activated Akt has critical roles in cellular processes, such as apoptosis, cell survival, and cell progression, as well as T2DM pathogenesis (10).

Activated Akt, which is primarily expressed in insulin-responsive tissues, promotes glucose transporter 4 (GLUT4) translation (14). Akt possibly influences insulin signal transmission and glucose transport (15). Insulin increases glucose uptake in cells by stimulating GLUT4 translocation from the intracellular sites to the cell surface; moreover, up to 75% of insulin-dependent glucose disposal occurs in skeletal muscle (15). Reducing *GLUT4* expression and translocation in a cell may result reduced glucose uptake and thus cause insulin resistance (16). Mulberry leaf extract stimulates glucose uptake and GLUT4 translocation to the plasma membrane of adipocytes through the PI3K-mediated signaling pathway (17). A flavonoid isolated from rutin enhances insulin-dependent receptor kinase activity and GLUT4 translocation in differentiated muscle myotubes and thereby improves glucose uptake (18). Mango leaf extract affects glucose and lipid homeostasis *in vitro* and *in vivo* through the PI3K/Akt and Adenosine monophosphate-activated protein kinase (AMPK) signaling pathways (19). Bamboo leaf extract treatment could increase the phosphorylated Akt level in renal tissues of rats with diabetes (20). Therefore, insulin-like activity, such as the stimulation of glucose uptake by skeletal muscle through PI3K/Akt pathways, may be crucial in regulating blood glucose level.

White sweet potato (WSP; *Ipomoea batatas* L.) belongs to the Convolvulaceae family. WSP extracts have antidiabetic activity in both insulin-deficient and -resistant diabetic models (21–25). In patients with T2DM, WSP tuber extract effectively reduced insulin resistance as well as fibrinogen, fasting plasma glucose, and low-density lipoprotein-cholesterol levels (26–28). In our previous clinical trial, meal replacement therapy using whole tuber of WSP Tainung No. 10 (TNG10) – a new WSP cultivar that can provide 15.5 g of fiber per 100 g and has an average glycemic index of 36.2 – was found to reduce energy and glucose absorption in the intestines (29). WSP incorporated into enteral formulas also can improve nutrition status and glycemic control in elderly diabetic patients (30).

Thus far, animal studies on the use of native WSP tubers (WSP-T) or leaves (WSP-L) as a functional ingredient for the management of non-insulin-dependent diabetic mice have been scant. This study thus evaluated the effects of various WSP-T or WSP-L dosages on antidiabetic activity involving PI3K/Akt pathway activation in mice with STZ–NA-induced diabetes. These results may provide insights into the use of WSP as a potential functional food for treating T2DM. Moreover, the influence of WSP on islet function and morphology was investigated.

## Materials and methods

### Plant materials

Fresh mature *I. batatas* L. TNG10, a starch-rich WSP variety, were harvested from a farm in the Chiayi Agricultural Experiment Station, Taiwan. The WSP TNG10 tuber were first washed and then sliced (thickness: 3–5 mm). The WSP leaves were washed and air-dried. Both sliced sweet potatoes and treated leaves were lyophilized and ground using 200 mesh (75 μm) for use in animal diet.

### Experimental design and treatment schedule

Male Institute of Cancer Research (ICR) mice (*n* = 30, age: 4 weeks) were obtained from BioLASCO Taiwan (Taipei, Taiwan). Taipei Medical University approved the use of these laboratory animals (LAC-100-0202). The mice were housed throughout the feeding experiment in a room maintained on a 12-h light–dark cycle at a constant temperature of 24°C with relative humidity of 65 ± 15%. They were allowed free access to food and water and were fed the American Institute of Nutrition (AIN)-93G (31). After 2 weeks of adaptation, diabetes mellitus (DM) was induced in the mice by two intraperitoneal injections of NA (120 mg/kg body weight [b.w.]) plus STZ 50 mg/kg b.w.; Sigma, Saint Louis, MO, USA). NA, dissolved in saline, was injected intraperitoneally 15 min before the administration of STZ, which was freshly dissolved in citrate buffer (pH 4.5) to induce diabetes at 1-day interval (9, 32). Normal saline (0.9% NaCl) was used as a vehicle injection for the normal control (NC) group. During the experimental period, the animals’ food intake and body weight were monitored once a week. A mouse was considered hyperglycemic when its fasting plasma glucose concentration was > 180 mg/dL at 2 weeks after the last induction date. The mice were divided into six experimental groups comprising five animals each: NC, DM, DM plus 10% tuber (DM+10%-T), DM plus 30% tuber (DM+30%-T), DM plus 0.5% leaf (DM+0.5%-L), and DM plus 5% leaf (DM+5%-L). The compositions of the experimental diets are detailed in Table 1. Both NC and DM groups received an AIN-93G diet. The 10%-T and 30%-T groups received an AIN-93G diet containing 100 or 300 g/kg of WSP-T. Moreover, the 0.5%-L and 10%-L groups received an AIN-93G diet containing 5 or 50 g/kg of WSP-L, respectively. All experimental diets were equal in terms of calories (4.16 calories per gram), equal nutrients composition (carbohydrate: protein: fat = 63.5%: 20.5%: 16%), and fibers to ensure that no blood marker changes due to energy or cellulose imbalance occurred.

**Table 1:** Percentage composition of the experimental diet

Content (g/kg)	Diets				
	Normal control (NC)/diabetes mellitus (DM)	DM + 10%-T (10% white sweet potato [WSP] tuber)	DM + 30%-T (30% WSP tuber)	DM + 0.5%-L (0.5% WSP leaf)	DM + 5%-L (5% WSP leaf)
Powdered tubers	-	100	300	-	-
Powdered leaves	-	-	-	5	50
Corn starch	397.4	338.6	220.9	397.4	388.4
Casein	200	195.1	185.3	199	189.5
Dextrinized cornstarch	132	132	132	132	132
Sucrose	100	94.8	84.4	100	100
Soybean oil	70	70	70	70	70
Fiber	50	42.7	28.2	48	30
AIN-93 mineral mix	35	35	35	35	35
AIN-93-vitamin mix	10	10	10	10	10
L-Cysteine	3	3	3	3	3
Choline bitartrate	2.5	2.5	2.5	2.5	2.5
Tert-butylhydroquinone (mg/kg)	0.014	0.014	0.014	0.014	0.014

Each experimental chow diet has equal calories (4.16 calories per g) and equal nutrients composition (carbohydrate: protein: fat = 63.5%: 20.5%: 16%).

### Blood markers

A glucose tolerance test was conducted before the completion of the experiment. The mice were starved overnight, and the blood samples were taken from the tail vein (time 0). A glucose challenge was given (1 g glucose/kg b.w.), and other blood samples were obtained after 30, 60, 90, and 120 min by tail vein sampling (33). Plasma glucose concentrations were determined; the area under the curve (AUC) for blood glucose was also calculated (34). After 8 weeks of these diets, the mice were starved overnight and then anesthetized in their sleep with isoflurane. Blood was collected in non-anticoagulant tubes and tubes containing ethylenediaminetetraacetic acid. Serum and plasma were prepared and stored at −20°C for further insulin and lipid measurement. Blood was also collected in tubes containing heparin, and plasma was prepared and stored at −20°C for glucose measurement. The homeostatic model assessment-insulin resistance (HOMA-IR) index was calculated as (fasting plasma glucose × plasma insulin/22.5) to assess insulin resistance (35). Plasma insulin levels were measured using a mouse insulin enzyme-linked immunosorbent assay kit (Mercodia, Uppsala, Sweden). Tumor necrosis factor alpha (TNF-α) levels were determined by using an enzyme-linked immunosorbent assay (BioLegend, San Diego, CA, USA) according to the manufacturer’s instructions. The levels of other blood biomarkers, namely, alanine transaminase (ALT), triglyceride (TG), and total cholesterol (TC), were analyzed from a 0.1 mL serum sample in the National Laboratory Animal Center (Taipei, Taiwan).

### Western blotting

The gastrocnemius muscles were homogenized in a modified Radioimmunoprecipitation assay buffer (RIPA) buffer (0.5 M Tris–HCl at pH 7.4, 1.5 M sodium chloride, 2.5% deoxycholic acid, 10% NP-40, and 10 mM ethylenediaminetetraacetic acid) and 10% protease and phosphatase inhibitor cocktail (Sigma, Saint Louis, MO, USA). The homogenates were centrifuged at 10,000 g at 4°C for 15 min, and the supernatants were collected. For demonstrating GLUT4 expression in the membrane, the muscle lysate was prepared using a Mem-PER kit (Thermo Fisher Scientific, Waltham, MA, USA) to enrich the membrane proteins. Protein concentrations in each sample were quantified using a commercial assay kit (Bio-Rad DC Protein Assay kit, Bio-Rad Laboratories, Hercules, CA, USA) with bovine serum albumin as a standard. Equal amounts of proteins (40 μg) were denatured and separated through 10% sodium dodecyl sulfate polyacrylamide gel electrophoresis and then transferred onto a polyvinylidene difluoride transfer membrane (Amersham Biosciences, Little Chalfont, Bucks, UK). These blots were then incubated with primary antibodies, namely anti-IR (ab69508, WB 1:250; Abcam, MA, USA), anti-phosphorylated IR Thr1146 (p-IR-Thr1146, 3021, WB 1:1000; Cell signaling, MA, USA), anti-Akt (2938, WB 1:1000; Cell signaling, MA, USA), anti-phosphorylated Akt (p-Akt; 9018, WB 1:1000; Cell signaling, MA, USA), and anti-GLUT4 (ab654, WB 1:2000; Abcam) antibodies, at 4°C overnight and then with secondary antibodies, namely goat anti-rabbit or goat anti-mouse (Abcam) antibodies, at a 1:5000 ratio at room temperature for 1 h. The binding of antibodies was determined using FAST 5-bromo-4-chloro-3-indolyl phosphate/nitro blue tetrazolium as the substrate of the secondary antibody-conjugated alkaline phosphatase. The band density was quantified using the analysis software Quantity One 1-D (Bio-Rad, Hercules, CA, USA). All controls for Western blotting were applied to ensure antibody specificity and protein consistency.

### Pathological tissue preparation

The pancreas and liver tissues isolated from the sacrificed animals were fixed in 10% neutral buffered formalin solution, dehydrated by passing through a graded series of alcohol, and embedded in paraffin blocks; these blocks were then cut into 5-μm-thick sections by using a Leica RM 2245 rotary microtome (Leica Microsystems, Wetzlar, Germany). These sections were stained using hematoxylin and eosin (H&E). A pathologist blinded to the treatments performed the histological evaluation. The photomicrographs of each tissue section were observed on the EVOS FL Imaging System (Thermo Fisher Scientific, Waltham, MA, USA) and then analyzed using Image J (http://rsb.info.nih.gov/ij/) for area percentage.

### Statistical analysis

Numerical data are presented as means ± standard deviations. Statistical evaluation was performed using one-way analysis of variance (ANOVA), followed by Duncan’s multiple range test. All data analyses were performed using SPSS (version 19; SPSS Inc., Chicago, IL, USA). Differences were considered significant at *P* < 0.05.

## Results

### Body weight, food intake, and feed efficiency ratio

The body weight, weight gains, food intakes, and feed efficiency ratios (FERs) of the mice are presented in Table 2. The weight gain was significantly lower in the DM group than in other control groups (*P* < 0.05). The consumption of different dosage of tuber or leaf did not influence the final body weight and weight gain in diabetic mice fed the experimental diets. The FER was significantly higher in DM+10%-T, DM+30%-T, DM+0.5%-L mice and DM+5%-L than in the DM group (*P* < 0.05).

**Table 2:** Body weight, food intake, and feed efficiency ratio in the mice fed with experimental diets

Group	Normal control (NC)	Diabetes mellitus (DM)	Diabetes mellitus plus 10% tuber (DM + 10%-T)	Diabetes mellitus plus 30% tuber (DM + 30%-T)	Diabetes mellitus plus 0.5% leaf (DM + 0.5%-L)	Diabetes mellitus plus 5% leaf (DM + 5%-L)
Initial body weight (g)	33.4 ± 1.0	34.9 ± 1.5	34.7 ± 2.5	32.0 ± 2.0	36.0 ± 2.5	34.4 ± 3.2
Final body weight (g)	40.9 ± 2.4	39.4 ± 1.5	43.0 ± 3.6	43.3 ± 5.3	47.1 ± 6.9	41.8 ± 6.2
Weight gain (g/day)	0.152 ± 0.04	0.080 ± 0.020	0.180 ± 0.044^*^	0.202 ± 0.065^*^	0.198 ± 0.059^*^	0.192 ± 0.038^*^
Food intake (g/day)	4.50 ± 0.54	5.50 ± 0.95	5.11 ± 0.52	4.80 ± 0.86	4.70 ± 0.76	4.78 ± 0.85
Feed efficiency ratio (%)	3.22 ± 0.23	1.45 ± 0.16	3.52 ± 0.27*	4.21 ± 0.25*^a^	4.21 ± 0.22^*^	4.02 ± 0.28^*^

Asterisks indicate significance level compared to DM group. Letter ‘a’ indicates significance level to DM + 10%-T group. All values are presented as means ± standard deviation (*n* = 5). Statistical evaluation was performed using one-way ANOVA, followed by Duncan’s multiple range test, *P* < 0.05. Feed efficiency ratio (FER, %) = (Body weight gain (g/day)/food intake (g/day)) × 100.

### Biochemical findings

**Fig. 1:**
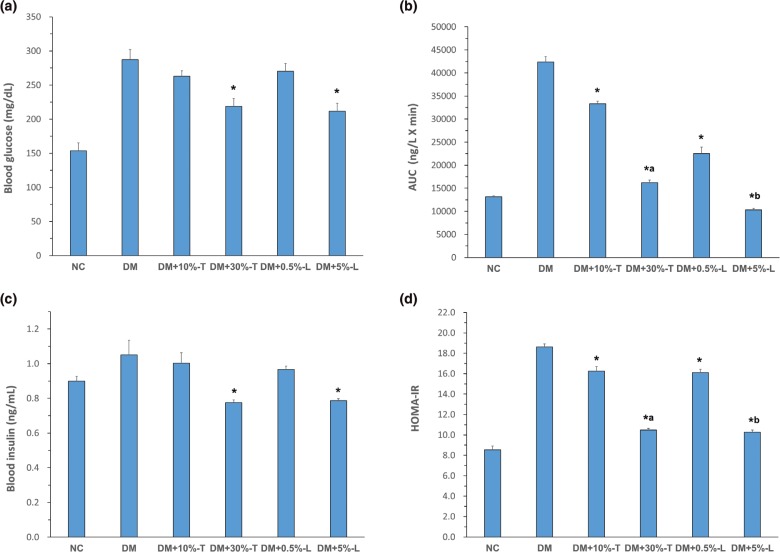
Blood glucose and insulin index analysis in various groups. (a) Fasting blood glucose concentration. (b) Glucose area under the curve (AUC 0–120 min). (c) Blood insulin level. (d) The homeostatic model assessment-insulin resistance (HOMA-IR) in various groups after 8 weeks of the white sweet potato intervention. Asterisks indicate significance level compared to DM group. Letter ‘a’ indicates significance level to DM + 10%-T group. Letter ‘b’ indicates significance level to DM + 0.5%-L group. All values are presented as means ± standard deviation (*n* = 5). Statistical evaluation was performed using one-way ANOVA, followed by Duncan’s multiple range test, *P* < 0.05.

**Fig. 2:**
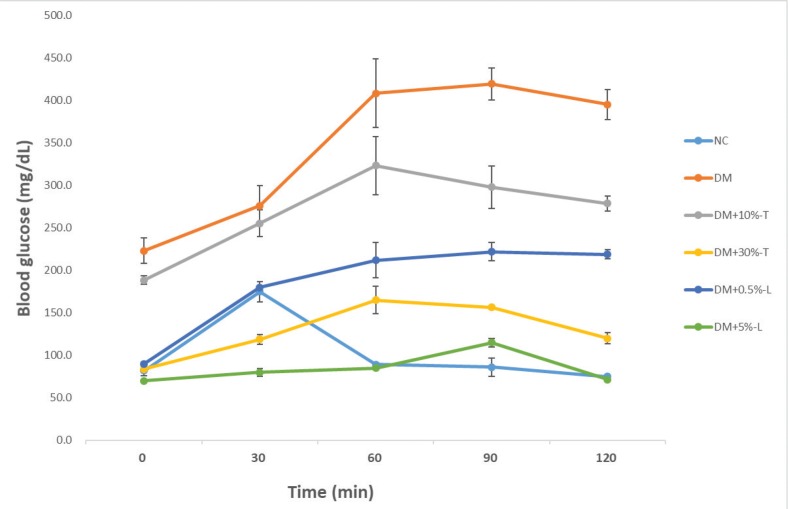
Glucose tolerance test in various experimental diet. All values are presented as means ± standard deviations (*n* = 5). Plasma blood glucose levels were determined in different time (0–120 min). NC, normal control; DM, diabetes mellitus; DM+10%-T, diabetes mellitus plus 10% tuber; DM+30%-T, diabetes mellitus plus 30% tuber; DM+0.5%-L, diabetes mellitus plus 0.5% leaf; DM+5%-L, diabetes mellitus plus 5% leaf.

After the 8-week intervention with different experimental diets, the groups exhibited no significant differences in the average body weight, organ weight, or feed efficiency. The mean fasting blood glucose was 153.8 ± 11.5 and 287.4 ± 14.4 mg/dL in the NC and DM mice, respectively. All mice with STZ–NA-induced diabetes exhibited mild hyperglycemia. The blood glucose level was 262.9 ± 7.9 and 270.1 ± 11.6 mg/dL in DM+10%-T or DM+0.5%-L mice, respectively, whereas it was 218.8 ± 11.5 and 211.6 ± 11.5 mg/dL in DM+30%-T or DM+5%-L mice, respectively. Thus, the higher the tuber (30%-T) or leaf (5%-L) dosage, the more was the pronounced reduction effect on fasting blood glucose levels, AUC and glucose tolerance test, compared with AIN-93G diet alone (Figs. 1a, b and 2). The AUC derived from the glucose tolerance test was next used to diagnose impaired glucose tolerance. Compared with that of DM mice (100%), the AUC of DM+5%-L, DM+0.5%-L, DM+30%-T, and DM+10%-T mice decreased to 24%, 53%, 38%, and 79%, respectively. Thus, both DM+5%-L and DM+30%-T mice demonstrated significant reduction in the AUC (Fig. 1b). After intervention, the plasma insulin levels slightly increased, by a factor of 0.71, in DM+5%-L and DM+30%-T mice (Fig. 1c). Moreover, HOMA-IR indicated significantly higher insulin resistance in DM mice than in the other groups. Similarly, compared with DM mice, HOMA-IR considerably improved in DM+10%-T (0.83-fold improvement), DM+30%-T (0.54-fold improvement), DM+0.5%-L (0.83-fold improvement), and DM+5%-L (0.53-fold improvement) mice (Fig. 1d). Taken together, these results indicate that our experimental diets with higher percentages of tuber or leaf from WSP improved the glycemic markers in the diabetic mice.

In mice, DM induction increased plasma ALT levels by up to 120.53 ± 45.96 U/L (Table 3). However, ALT decreased by a factor of 0.54 and 0.31 in DM+30%-T and DM+10%-T mice, respectively. Similarly, in DM+0.5%-L and DM+5%-L mice these decreases were by a factor of 0.37–0.43. DM caused TG levels to increase to 151.13 ± 47.57 mg/dL in DM mice; nevertheless, in WSP-T or WSP-L–treated mice, the TG levels reduced by a factor of 0.3–0.63. However, no significant intergroup differences were observed in the ALT or TG levels between the WSP-T- or WSP-L-administered mice. The TC also did not differ significantly among groups. DM mice exhibited increased levels of the inflammatory marker TNF-α (45.18 ± 5.72 pg/mL), which significantly decreased after intervention with higher dosage of WSP-T or WSP-L; this decrease was significant, by a factor of approximately 0.66, after intervention with 5%-L (29.86 ± 5.24 pg/mL) compared with the DM group.

**Table 3:** Mouse blood biochemistry test after 8 weeks of experimental diet intervention

Group	Normal control (NC)	Diabetes mellitus (DM)	Diabetes mellitus plus 10% tuber (DM + 10%-T)	Diabetes mellitus plus 30% tuber (DM + 30%-T)	Diabetes mellitus plus 0.5% leaf (DM + 0.5%-L)	Diabetes mellitus plus 5% leaf (DM + 5%-L)
Alanine transaminase (ALT) (U/L)	50.34 ± 15.85	120.53 ± 45.96	65.03 ± 22.74^*^	37.76 ± 7.41^*^	44.01 ± 12.76^*^	51.48 ± 11.68^*^
Triglyceride (TG) (mg/dL)	86.34 ± 23.46	151.13 ± 47.57	95.42 ± 30.85	72.44 ± 27.05^*^	45.13 ± 16.18^*^	53.50 ± 18.55^*^
Total cholesterol (TC) (mg/dL)	160.72 ± 44.62	158.98 ± 23.36	210.02 ± 44.08	146.76 ± 23.15	175.30 ± 41.86	148.62 ± 41.98
Tumor necrosis factor alpha (TNF-α) (pg/mL)	29.37 ± 8.64	45.18 ± 5.72	42.22 ± 8.12	39.48 ± 8.23^*^	43.11 ± 8.91	29.86 ± 5.24^*a^

Asterisks indicate significant difference compared to DM group. Letter ‘a’ indicates significance level to DM + 0.5%-L group. All values are presented as means ± standard deviations (*n* = 5). Statistical evaluation was performed using one-way ANOVA, followed by Duncan’s multiple range test, *P* < 0.05.

### Histological findings

**Fig. 3:**
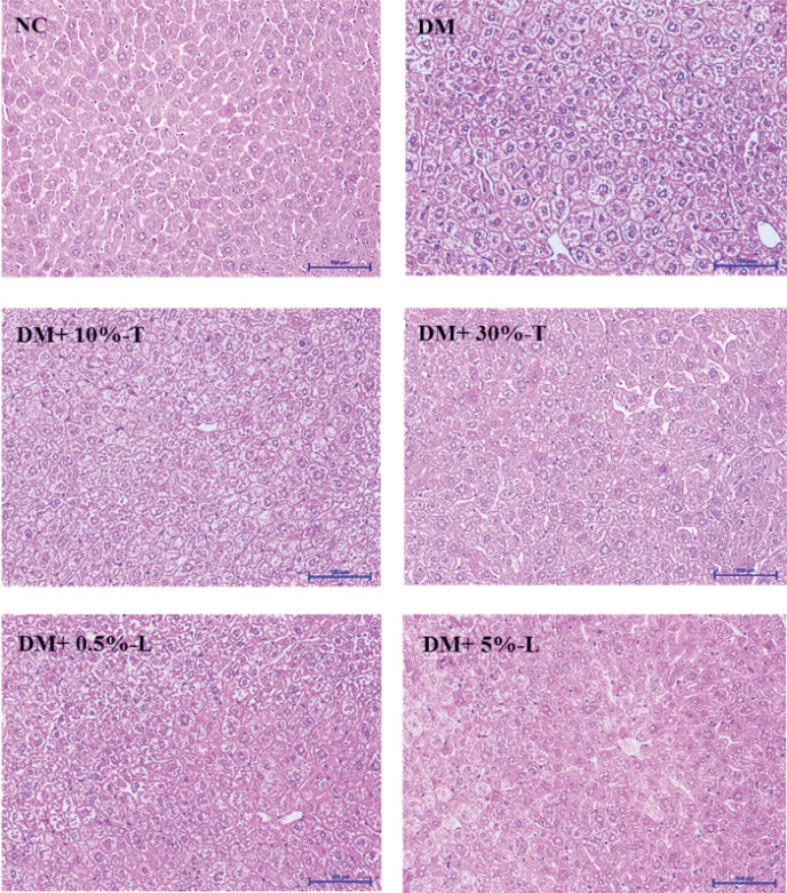
Histochemical characterizations in liver tissues stained using hematoxylin and eosin after various experimental diet interventions. Scale bar length represents 100 μm at 100× magnification. NC, normal control; DM, diabetes mellitus; DM+10%-T, diabetes mellitus plus 10% tuber; DM+30%-T, diabetes mellitus plus 30% tuber; DM+0.5%-L, diabetes mellitus plus 0.5% leaf; DM+5%-L, diabetes mellitus plus 5% leaf.

H&E staining of liver tissue section, observed at a 100× magnification, revealed that DM mice demonstrated greater liver cytoplasmic vacuoles and inflammatory cell infiltration compared with mice in the other groups. The different dosage of WSP-T or WSP-L used in DM mice has slight morphological or structural changes in the liver tissue (Fig. 3); however, the photomicrographs in different concentrations of WSP-T or WSP-L were difficult to quantify by pathology software analysis.

**Fig. 4:**
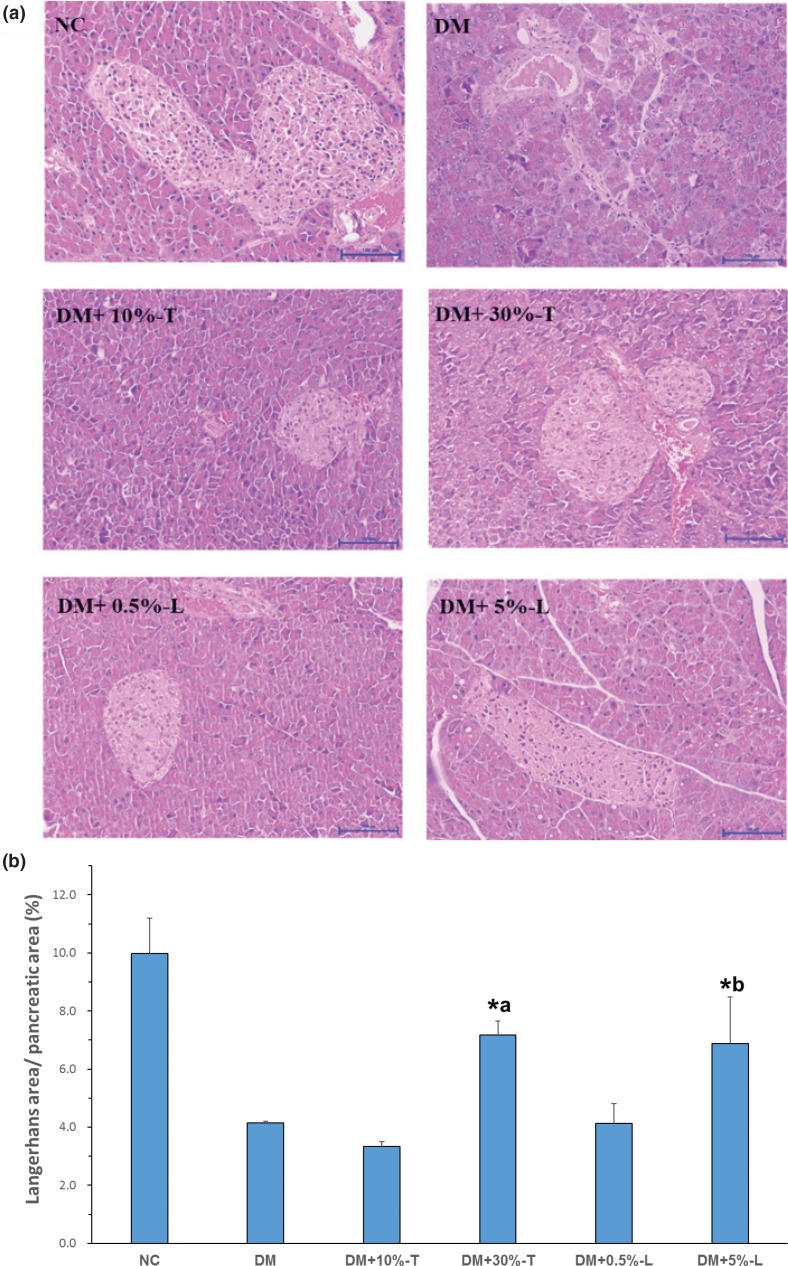
Histochemical characterizations in pancreatic tissues stained using hematoxylin and eosin (H&E) after various experimental diet interventions. Scale bar length represents 100 μm at 100× magnification. (a) Morphological changes in pancreatic islets were observed through H&E staining after the white sweet potato intervention. (b) The size of the Langerhans area in the photomicrographs was determined using ImageJ. Asterisks indicate significant difference compared to DM group. Letter ‘a’ indicates significance level to DM + 10%-T group. Letter ‘b’ indicates significance level to DM + 0.5%-L group. All values are presented as means ± standard deviations (*n* = 5). Statistical evaluation was performed using one-way ANOVA, followed by Duncan’s multiple range test, *P* < 0.05.

The histology of pancreatic islets was normal in the NC group. The H&E-stained histological sections of pancreas tissues of DM mice consistently revealed degenerative and necrotic changes and shrunken sections in the islets of Langerhans (Fig. 4). The nuclei of necrotic cells indicated pyknosis or marginal hyperchromasie. The results of semi-quantitative analysis from H&E staining are presented in Fig. 4b. The proportion of Langerhans’s area in the photomicrographs was calculated using ImageJ. Langerhans’s area was reduced by 60% in DM mice, but DM+30%-T or DM+5%-L mice demonstrated significantly restored Langerhans’s area by approximately 30%.

### Western blotting findings

**Fig. 5:**
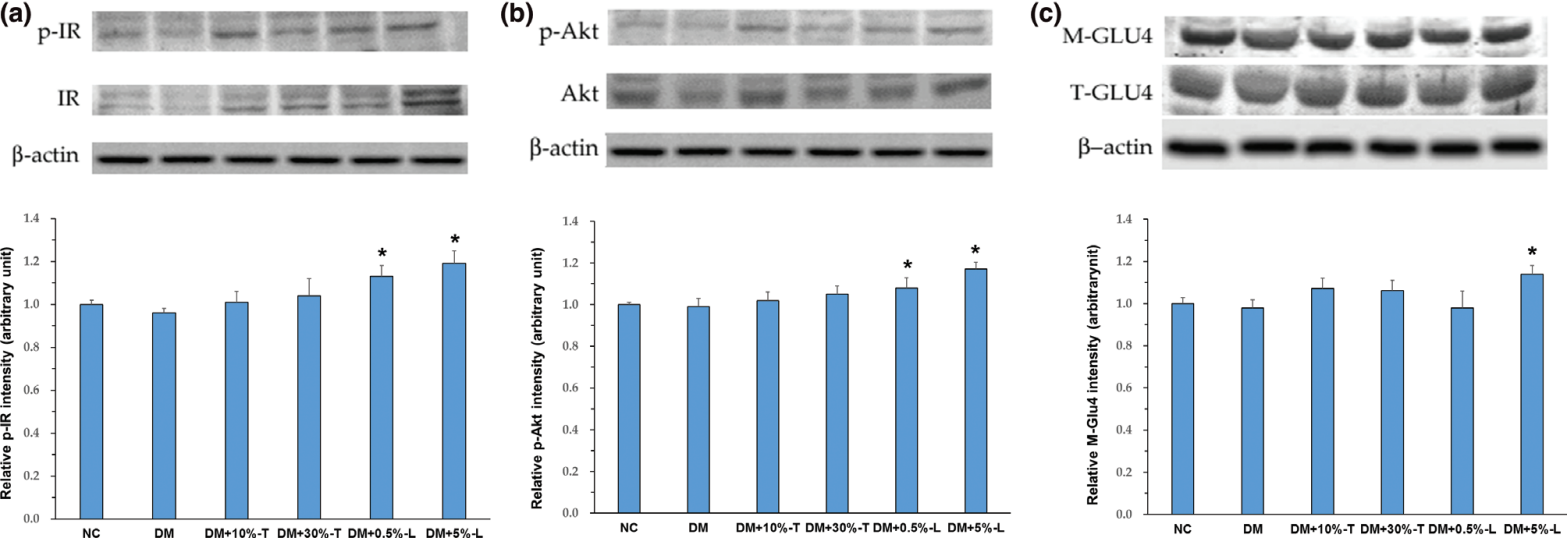
Expression of insulin-signaling-related proteins in the muscle. Protein expression of p-IR & IR (panel a), p-Akt & Akt (panel b), and M-GLUT4 & T-GLU4 (panel c) were analyzed with Western blotting of the homogenates in the gastrocnemius muscle. Data are expressed as means ± standard deviations (*n* = 5). p-IR, phospho-insulin receptor; IR, insulin receptor; p-Akt, phospho-protein kinase B; Akt, protein kinase B; M-GLU4, membrane glucose transporter 4; T-GLU4, total glucose transporter 4; Asterisks indicate significant difference compared to DM group. Statistical evaluation was performed using one-way ANOVA, followed by Duncan’s multiple range test, *P* < 0.05.

We then explored whether the antidiabetic activity of the WSP-T or WSP-L was involved in the PI3K/Akt pathway. Thus, we evaluated the expression of candidate proteins, such as phosphorylated and unphosphorylated IR and Akt, in the aforementioned pathway as well as translocation of GLUT4 vesicles from cytosol to the cell membrane in muscles (Fig. 5). No significant differences were observed in the expression of the three candidate proteins in NC and DM mice. No significant differences were noted even in DM+10%-T or DM+30%WSP-T mice. By contrast, compared with other groups, after 8 weeks of treatment, DM+5%-L mice demonstrated slight increases in the relative intensity of p-IR (by 24%), p-Akt (by 18%), and membrane GLUT4 (M-GLUT4; by 17%). Thus, higher WSP-L dosage partially increased insulin sensitivity possibly via the insulin-signaling pathway and reduce blood glucose levels in diabetic mice.

## Discussion

*I. batatas* L., also known as sweet potato with different varieties, is a valuable medicinal plant for the anticancer, antidiabetic, and anti-inflammatory activities in its extract (36, 37). In this study, mouse blood biochemistry indicated increased ALT, TG, and TNF-α levels resulting from increased free radical production caused by STZ in diabetic mice; however, these levels were significantly lower in mice who received the WSP-T or WSP-L intervention for 8 weeks. WSP leaves are rich in potent phytochemicals that can fight free radicals, and their roots are rich in dietary fiber; antioxidants and vitamins in them can also capture free radicals (36). Nevertheless, WSP-bioactive ingredients reduce not only blood glucose and insulin levels but also insulin resistance by improving the HOMA-IR index. In the preclinical phase of T2DM, insulin resistance is initially compensated for by increased insulin secretion; however, this prolonged overstimulation of insulin secretion causes the gradual failure of β-cells over time (38, 39). Therefore, in this study, Langerhans’s area was significantly reduced in the diabetic group, but was significantly recovered by the 30% WSP-T or 5% WSP-L intervention. The antidiabetic activity of WSP is partly due to the regeneration of pancreatic islets, which lowers the blood glucose level and its AUC. Our results are consistent with those of Sunarti et al. (40): pancreatic β-cell regeneration by white-skinned sweet potato (WSSP) in rats with STZ-induced diabetes might increase the number and size of islets and thus result in the formation of small new islets adjacent to the duct in pancreatic tissue.

In WSP roots or leaves, the major phytochemicals are flavonoids, terpenoids, tannins, saponins, glycosides, alkaloids, steroids, and phenolic acids (37, 41). Some medical plants are associated with regeneration of the Langerhans’s area, and phytochemicals are thus used for the treatment of diabetes. The epigallocatechin gallate intervention can moderate the decrease in the islet mass induced by multiple low doses of STZ in mice (42). The flavonoid-rich fraction of *Pilea microphylla* can preserve the islet architecture and prevent hepatocyte hypertrophy based on the histopathology of the pancreas and liver in high-fat mice with STZ-induced diabetes (43). However, all WSP phenolic compounds responsible for the differences in the results warrant biological metabolite analysis to clarify blood sugar-lowering mechanisms.

The insulin-stimulated glucose uptake in skeletal muscle plays a major role in regulating glucose metabolism and energy homeostasis. Therefore, the targets of the PI3K/Akt pathway are critical because treatments of T2DM function involve the impairment of this signaling pathway in muscles, all of which causes hyperglycemia and insulin resistance (10, 15). Bae et al. reported that mulberry leaf extract reduced fasting blood glucose and insulin levels, enhanced insulin sensitivity, and increased p-Akt and p-AMPK expression in db/db mice (44). The hypoglycemic effect of *Myrcia bella* leaf extract in mice with STZ-induced diabetes can increase the expression of the insulin-signaling pathway involving PI3K/Akt proteins (45). In the current study, Western blotting indicated that 5% WSP-L slightly increased the p-IR, p-Akt, and M-GLUT4 expression, we speculate that the direct use of lyophilized and ground WSP-L may not effectively release biologically active substances, resulting in an insignificant PI3K/AKT signal expression in mice muscle tissue. In addition, AMPK-mediated hyperglycemia to insulin signal transduction was not present in our data, which needs more evidence to complete the antidiabetic mechanism by WSP-L.

According to Oki et al. (24), arabinogalactan isolated from the tuberous cortex of WSSP is an antidiabetic compound that can decrease the elevation in plasma glucose levels by reducing insulin resistance. The active component isolated from WSSP has a high molecular weight (24). In addition, resistant starch may be a promising dietary fiber for the preventing or managing diabetes and the related diseases. In humans, RS improves insulin resistance after chronic feeding through a mechanism involving changes to both adipose tissue and muscle metabolism (46). Additionally, we employed *in vitro* enzyme-digested WSP-T, which contained 0.36 ± 0.05 resistant starch and 48.0 ± 1.8 mg/100 mg slowly digestible starch (29). Therefore, the tuber active substances with antidiabetic effects, in relation to *in vivo* digestibility and glucose release rate, warrant an additional study.

## Conclusions

Insulin resistance and pancreatic islet dysfunction are the two features of T2DM. In this study, high dosage of WSP-T or WSP-L could significantly reduce fasting blood glucose levels, improving fasting glucose tolerance, lowering HOMA-IR, and regenerating pancreatic islets. Notably, among all concentrations of WSP-T or WSP-L used, the antidiabetic effects of a 5% WSP-L may be slightly better in terms of insulin-signaling pathway activation. Finally, this study confirmed that whole WSP can be used to treat T2DM, by using experiments on mice with diabetes, biochemical analysis, histomorphometry, and insulin-signaling pathway analysis. Our results indicate that the hypoglycemic effect in different concentrations of WSP-T or WSP-L can be used as raw materials or materials for health food manufacturing in the food industry.
